# The Future of Pediatric Urology

**DOI:** 10.3389/fped.2019.00100

**Published:** 2019-03-29

**Authors:** Philip Ransley

**Affiliations:** Department of Paediatric Urology, Sindh Institute of Urology and Transplantation, Karachi, Pakistan

**Keywords:** pediatric urology, advances, future, organization, global health

It is a slightly prejudiced view from the inside, but there seem to be few specialities which encompass such a wide and divergent range of conditions that we in pediatric urology are faced with almost every day of our working lives. As a consequence, we are exposed to and have to accommodate advances in many different areas of medicine from endocrine treatment of the fetus to exploring the limits of robotic surgery. I am not sure of the wisdom of asking a long retired pediatric urologist to speculate on the future of the speciality but then no-one can look forward with any certainty. In some ways we are programmed to resist change and sometimes we are just not able to grasp the significance of what we are being told. It is with some personal embarrassment that I recall reading the very first papers describing the theoretical possibility of extracorporeal shockwave therapy for renal stones and thinking to myself that “this can never catch on”! However, I am in good (or bad!) company. The embattled resistance to Lister's antiseptic technique by much of the profession for so many years reflects a corporate blindness which we still potentially suffer from today although modern communication alleviates this to some degree. In many ways a professional lifetime is like a journey in a fog. We have limited vision. The way ahead is shrouded in mist and far from clear but slowly appears as one moves forward while what we experienced in the past is swallowed and lost in the mists of time.

At the basic level of our surgical lives is surgical technique which has undergone steady refinement over centuries and decades with the occasional boost given by a conceptual breakthrough. The steady improvement in results from advances in suture material was a passive benefit but the conceptual advance of enlarging the bladder using bowel was ahead of its time and doomed to failure until the game-changing process of intermittent catheterization came to its rescue. I can remember reading the notes of a patient who had a colocystoplasty in 1966, less than a decade before CIC was introduced and became widespread. The anticipation and optimism shone through in the early pages but the dismal outcome and evolution to a classical conduit was equally well documented. That is the problem about trying to see the future. Some developments are the result of dogged years of background work, like transplantation, while others blossom from something not visualized before like ESWL or CIC. And yet, our innovative surgeon couldn't see what lay ahead, however simple. That is how I feel as I write. How can I introduce you to the things I cannot see!

The Mitrofanoff channel was another of those eureka moments and one has to say that the combination of sutures, CIC and a Mitrofanoff conspired to revolutionize our current practice of reconstructive pediatric urology. But if that was our lunar landing, we still yearn for Mars.

The advances in tissue engineering and command of stem cell differentiation may yet deliver for us the tissues that we long for. We know that the progress is slow, and the practical difficulties are great, but we can feel that an alternative to the use of bowel in the urinary tract is on the horizon and perhaps direct urethral substitution will become a possibility, but it is not here and now nor any time soon. It is with this in mind and with my future blindness that I have always tried to leave healthy, if non-functional, tissue behind in case it may be valuable as a cellular source in years to come. However, it is likely that in a manner similar to tissue created in culture for the treatment of burns, stem cells may be able to deliver small quantities of homologous tissue grown in the lab for reconstruction by conventional techniques. I am thinking of urethral reconstruction in the hypospadias cripple as the commonest challenge that would benefit greatly from this tiny incremental progress. It will be expensive and labor intensive, but it may open the door to a new world where tissue availability is no longer an issue. But it is others, not us at the coal face who will make the essential progress that we can put to good use.

The analysis of the human genome was released with a fanfare that promised much and genetic studies continue to probe the factors underlying everything we see and do. For a surgical simpleton like myself I thought only in terms of understanding congenital anomalies and although candidate genes have been identified for some conditions, the way to exploit such knowledge is not clear. However, the functional implications of genetic abnormalities are much more important such as the vulnerability to urinary infection or the development of embryonal tumors. If, as has been suggested, Wilms' tumor develops due to low activity of a gene responsible for maturing kidney cells, and that gene could be activated to suppress the tumor, we enter a magical world beyond my understanding.

The world of transplantation has grown enormously since the first renal transplant more than half a century ago and thanks to the sophisticated advances in immunosuppression has taken on an almost routine nature. The expansion of this world into liver, face and limbs has little impact on pediatric urology but what about the uterus and genitalia? It is early days as yet, but the first baby from a fertilized egg implanted into a transplanted uterus has been born. Far from a routine, what happens if it becomes more widely applicable? In circumstances where potential fertility is part of the assessment and decisional judgment, will we have to consider potential uterine transplantation on one side of the equation? War is recognized as a great stimulant to innovation and sadly landmine injuries took their toll of external genitalia which is now being addressed by genital transplantation. Will that too cascade down into pediatric urology and be part of our discussion in very severe genital anomalies?

Technology surrounds us in everyday life and artificial intelligence beckons. We are living in a world where simple thoughts can be transferred from one individual to another when both are wearing EEG caps and the signals are analyzed by AI. If only we could communicate with our trainees that way! But maybe AI can be trained to recognize a full bladder and take whatever action is appropriate. Combining technology with stem cell expertise is already promising an implantable kidney whereby sheets of differentiated renal tubular cells are perfused by blood. It will be a shame if such spectacular progress is thwarted by the difficulties of transporting the “urine” produced to the bladder in a conduit of artificial material.

Of course, on a more prosaic level the technological advance which impacts all of us in everyday life is the development of robotic assisted surgery. We are all familiar with its potential and its boundaries are being extended almost daily. A new skill set is required which the younger generation are completely at home with, but we have not yet exploited the very reason that the system was developed which is remote surgical intervention which would challenge some of our classical understanding of responsibility in surgical intervention. Similarly, we may be challenged by the idea of a robot technician (still a human one as far as my myopic vision goes) who is trained to do some repetitive tasks well such as suturing a long anastomosis.

All of these amazing prospects are very largely dependent on the investment, skills and commitment of others outside our world of pediatric urology with whom we collaborate, ready to bring their advances “to the table” when the time is right. We can do little to assist their progress but maintain a dialog and be ready when the time comes. This should cause us to take a good look at ourselves and whether we are in the best condition possible to face what the future brings. The one thing that is clear in my mind is that the future of pediatric urology lies in some reorganization of our structure. First of all, we need to look at what has happened to pediatric urology. From tentative early beginnings pediatric urology has expanded enormously and independent units have grown up in almost every university hospital. There are now over 1,000 fellowship trained pediatric urologists in the United States and when the British Association of Pediatric Urologists was founded the 10 members could sit round a table. At the last meeting I went to there were, I think, more than 70 people. A combined European and American pediatric urology meeting can now attract over 1,000 delegates. The early pioneers in the speciality would be flabbergasted. In some ways this is wonderful news and parents can bring their children to a specialist with ease. However, this expansion has coincided with a significant change in our potential workload, at least in the western world. I have to say that my comments are really based on the experience and demographics of the western hemisphere although heavily influenced by two decades of experience in south Asia.

We have built the fundamentals of a pediatric urological service on expertise acquired from treating large numbers of patients with a limited number of conditions. Numbers are important whether it be for maintaining a personal level of expertise or training the next generation and the less common the problem the more important numbers become. There are a limited number of conditions in our speciality such as the Exstrophy/Epispadias complex, the cloacal anomaly spectrum, bilateral single ectopic ureters and the prune belly syndrome which require huge experience and immense technical skill to maximize the quality of life with minimal intervention. On reflection, I would include the ectopic ureterocoele in this list. The prolonged suffering of these unfortunate children through serial unsuccessful interventions is sadly still a common occurrence today. These are conditions which for the moment depend entirely on our collective experience and individual surgical skill. Although intense studies continue into the genetic or epigenetic background to these conditions, we cannot see any preventative solution arriving any time soon and the need to maintain our skill levels remains paramount. Even relatively common conditions such as posterior urethral valves require the in-depth resources of an expert unit to manage them well as they present management challenges from so many different facets of the stressed urinary tract. Managing a high urine volume from a dilated upper tract draining into a bladder which may change from being of low volume, poorly compliant and hypercontractile to being an acontractile floppy bag requires an understanding which comes only with the experience of large numbers, and the crises are intensified as transplantation looms.

Unfortunately, the expansion of our speciality in terms of centers as well as individuals has coincided with a downward trend in the numbers of patients with serious congenital anomalies. One of the simple factors is simply the declining birth rate. Whatever the social or economic factors are in driving this trend, it is a simple fact that birth rates have almost halved in the last 5 decades following the post-war boom. My generation was privileged to witness and participate in the introduction of pre-natal diagnosis, another example of progress in which we were the recipients rather than the instigators of progress and honestly, I believe that we were not active enough in entering the prenatal world in the early days. This is changing now as the pediatric urologist brings the technological advances of miniaturization of endoscopic instruments and laser fibers to the field. Prenatal diagnosis was a revelation and we are still only beginning to understand the kaleidoscope of events which can ultimately lead to the post-natal state with which we are familiar. On thing which I can see through the fog is that in a generation or two we will have a better handle on intra-uterine obstruction and the benefits (or not) of intervention on renal or bladder outcomes. However, in the meantime prenatal diagnosis has brought with it the two-edged benefit of termination. Not surprisingly this is a contentious issue with many conflicting personal, religious and political philosophies but the fact is that termination has added to the reduction in severe structural anomalies entering the postnatal world. The high termination rate for trisomy 21 and severe cardiac anomalies automatically reduces the number of patients with associated genito-urinary anomalies. The urologically specific termination of severe outflow obstruction or structural abnormality such as cloacal exstrophy, is more limited due to the lack of specificity, but nevertheless has brought about a significant drop in the number of cases requiring the post-natal expertise a pediatric urologist can bring.

It is clear to me that this combination of rapid expansion and reduced complex workload is a lethal cocktail for the maintenance of standards, surgical advancement, or training of the next generation. Complex pediatric urology can no longer be spread thinly, dictated by the randomness of populations but for the future we will have to accept and embrace wholeheartedly the concept of centralization of expertise for specific conditions. Without this we will start going backwards. Some centralization occurs serendipitously perhaps dictated by geography or politics but one of the first efforts to do this was undertaken in the United Kingdom in the ‘nineties for the management of the Exstrophy/Epispadias complex so that only two centers in England and Wales are responsible for these conditions. It didn't happen overnight but came after several years of debate between the members of the British Association of Pediatric Urologists. Once a professional accord had been reached, the decision was taken to the Ministry of health and activated. This, of course, was relatively easy to do within a nationalized health system but the important principle was that this had been a professional decision communicated to the administration. This is how it should be, and I fear that if we do not acknowledge the changes that are occurring it will be the administrations that will in time impose their own decisions. There have been other attempts to address the same problems by other means and avoid what some see as the inverse stigma associated with centralization. One of these was the consortium convened in the eastern United States where the patients remained fixed but the surgeons from three institutions traveled in order pool and gain experience collectively. It works and has many benefits, but it is administratively cumbersome and insanely expensive. It is good that it is happening even if only to highlight the need which has brought it into being.

In some ways pediatric urology is following the trends seen in adult urology. The changes in surgical practice in one of our parent specialities has given rise to the situation in which the majority of urological workload is outpatient based. In Pediatric urology the rise in the investigation and treatment of bladder dysfunction, the influx of large numbers of cases detected by prenatal ultrasound but not requiring surgical intervention and the demise in surgical intervention for reflux combined with the reduction in complex anomalies has given rise to a similar situation. I am reminded of an occasion experienced while performing an open partial nephrectomy for a renal tumor as a visiting surgeon. One of the observers was an urology resident who had come to see it partly because he said that in his 4 years of urological residency, he had yet to see a kidney other than through a telescope! Life changes but our speciality needs to maintain a core of advanced surgical skills.

Our profession already accepts centralization for complex problems. Pediatric cardiac surgery, Liver transplantation, rare tumors, and fetal intervention are already areas which are centralized in many parts of the world. We have enjoyed an explosion in interest and expertise in pediatric urology but now is the time to reflect and consolidate our position. The hub-and -spoke model seems to fit our needs admirably. Consultations and routine interventions can take place locally to the benefit of young families, but the complex material is transferred centrally within the same team. Such a system concentrates data, provides for surgical mentorship and the development of a hierarchy for the training and development of the young surgeon while maintaining expertise through numbers. It is a win-win situation.

It is possible that even with a hub and spoke system each center may not receive enough cases to maintain expertise. It has become the authors firm conviction that we need to begin to see pediatric urology as a global cause, a global family. This feeling comes about from the privilege I have had of working in Pakistan over the last 20 years. This collaboration began in the classical way as a visiting fireman but as the years went by the relationship changed and I was able to draw on their enormous workload and experience. I can honestly say that I was a much more experienced and arguably a better reconstructive surgeon after 10 years of working in Pakistan three times a year than I was at the end of 30 years of full-time experience in London. The populations of many parts of the world including most of Asia, Africa and S. America have not seen the dramatic reductions in birth rate experienced in the West and prenatal diagnosis has not yet had any significant impact in many places. If, at this moment the expertise lies in the West but the babies who need that expertise are in the East then the conclusion is obvious, and it will be important to act before that expertise declines. As I peer through the fog, I can see vague shapes of the future coming into view. Every major center of Pediatric Urology in the West has established a formal link with a similar institution for mutual benefit. Each brings different dishes to the feast. On one side there are centuries of academic discipline, modern surgical development and rigorous training. They are complemented by patient numbers, new challenges and experience. Of course, any such system has to be mutually beneficial but that is not too difficult to envisage although it will require some change in attitude both on the part of our profession and those who administer us. However, the opportunities it opens up for training in both the routine and the exotic and the self-development of the experienced surgeon are truly wonderful. It is likely that any pediatric surgical sub-speciality will have to adopt the same course of action. Many institutions have taken the first tentative steps in this direction, but our campaign has to be to make it mainstream.

If, in desperation, I turn to radar to peer ahead, I can envisage a cure for cancer. I can see artificial blood. I can imagine that I see an implantable artificial kidney. I am sure that I recognize miniaturized robotic instruments controlled by AI and I think I can see tissue engineering coming to the aid of my grandchildren. My surgical life has contributed nothing to this progress but what I see clearly now is that experience is essential for expertise in a surgical speciality and that the future of pediatric urology lies in worldwide cooperation and that now is the time to begin making it happen.

## Author Contributions

The author confirms being the sole contributor of this work and has approved it for publication.

### Conflict of Interest Statement

The author declares that the research was conducted in the absence of any commercial or financial relationships that could be construed as a potential conflict of interest.

